# Supplementary material for: Splice-Junction-Based Mapping of Alternative Isoforms in the Human Proteome
Source: Cell Rep. Author manuscript; Available in PMC 2020 Jan 15. (PMC6961840; doi:10.1016/j.celrep.2019.11.026)
Supplement: 2 [file NIHMS1546469-supplement-2.zip › DF1/PXD009737/TestisLysC/TestisLysC_39_WNK1_FQPVSMAAPTAITEAGTQPQK.pdf]

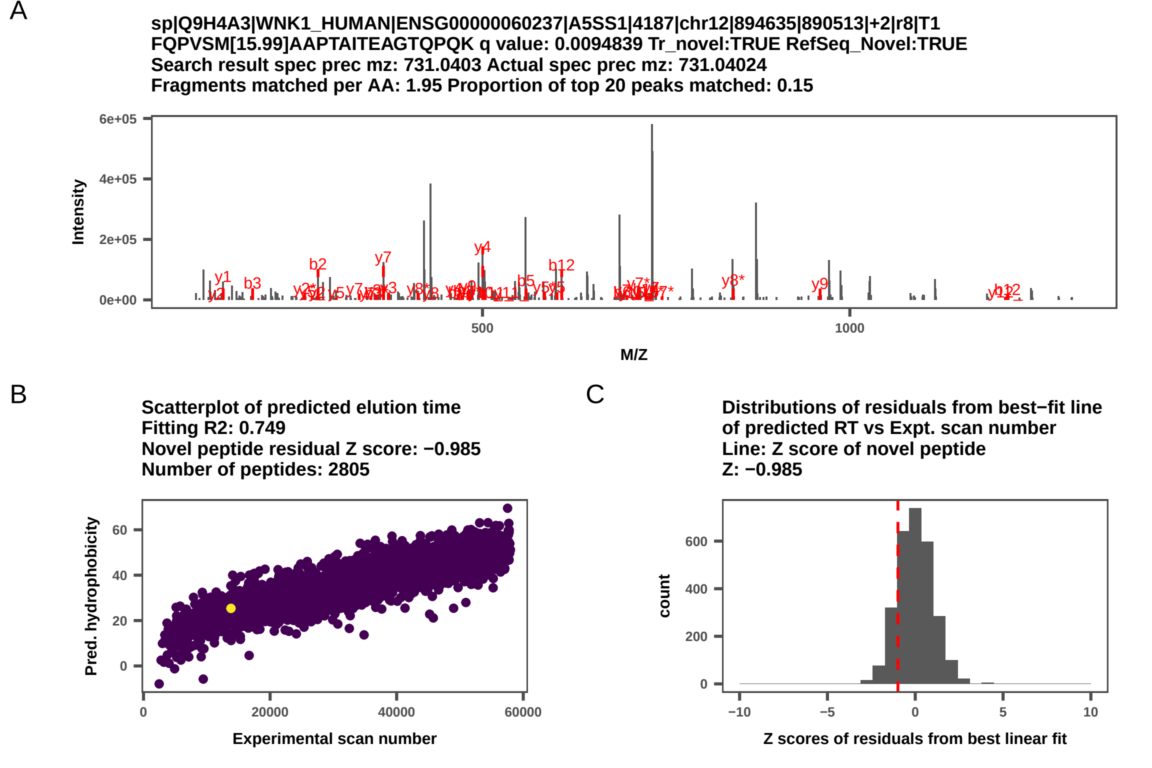
A
sp|Q9H4A3|WNK1_HUMAN|ENSG00000060237|A5SS1|4187|chr12|894635|890513|+2|r8|T1
FQPVSM[15.99]AAPTAITEAGTQPQK q value: 0.0094839 Tr_novel:TRUE RefSeq_Novel:TRUE
Search result spec prec mz: 731.0403 Actual spec prec mz: 731.04024
Fragments matched per AA: 1.95 Proportion of top 20 peaks matched: 0.15
Intensity
6e+05
4e+05
2e+05
0e+00
y1
b3
b2
y7
y4
b12
b5
y8*
y9
M/Z
500
1000
B
Scatterplot of predicted elution time
Fitting R2: 0.749
Novel peptide residual Z score: −0.985
Number of peptides: 2805
Pred. hydrophobicity
60
40
20
0
0
20000
40000
60000
Experimental scan number
C
Distributions of residuals from best−fit line
of predicted RT vs Expt. scan number
Line: Z score of novel peptide
Z: −0.985
count
600
400
200
0
−10
−5
0
5
10
Z scores of residuals from best linear fit
